# Safety and quality assessment of street‐vended barbecue chicken samples from Faisalabad, Pakistan

**DOI:** 10.1002/fsn3.3127

**Published:** 2022-11-12

**Authors:** Ayesha Ali, Naveed Ahmad, Atif Liaqat, Muhammad Adil Farooq, Samreen Ahsan, Muhammad Farhan Jahangir Chughtai, Abdul Rahaman, Kanza Saeed, Syed junaid‐ur‐Rahman, Azhari Siddeeg

**Affiliations:** ^1^ Institute of Food Science & Technology, Khwaja Fareed University of Engineering & Information Technology Rahim Yar Khan Pakistan; ^2^ National Institute of Food Science & Technology, University of Agriculture Faisalabad Pakistan; ^3^ School of Food Science and Engineering South China University of Technology Guangzhou China; ^4^ School of Food Science and Engineering Foshan University Foshan China; ^5^ Overseas Expertise Introduction Centre for Discipline Innovation of Food Nutrition and Human Health (111 Centre) Guangzhou China; ^6^ Department of Food Engineering and Technology University of Gezira Wad Medani Sudan

**Keywords:** barbecue chicken, heavy metals, microbiological safety, quality, unhygienic processing

## Abstract

The current study was designed to assess the safety and quality status of street‐vended barbecue chicken samples. The samples were collected from four regions of Faisalabad city: Ghulam Mohammad Abad (R_1_), Jhang Road (R_2_), Sargodha Road (R_3_), and Satiana Road (R_4_); and compared with the self‐prepared barbecue chicken sample (R_0_). Purposely, all the collected samples were subjected to assess the quality aspects by physicochemical analyses. The results of the physicochemical analysis showed that moisture content varied from 54% to 60%, crude protein 26.97% to 32.87%, crude fat 7.25% to 9.00%, crude ash 1.61% to 1.72%, pH 5.60 to 6.30, free fatty acid value 1.00% to 1.39%, and peroxide value 0.63 to 0.84 meq/Kg. Results pertaining to the enumeration of total microbial load and total coliform count exhibit 2.39–5.17 and 1.20–3.20 log cfu/g, respectively. The samples were assessed for heavy metals (Pb, Zn, Cd, and Fe) by atomic absorption spectrophotometer (AAS). The concentration of highly toxic metals Pb and Cd was found to be much higher than recommended value as they ranged from 1.90 to 3.70 mg/kg for Pb and 0.10 to 0.90 mg/kg for Cd. However, the level of essential metals (Fe and Zn) in barbecue chicken samples ranged from 67.10 to 180 and 8.30 to 35.80 mg/kg which was much higher than their safe limits for Fe (15 ppm) and Zn (5 ppm), respectively. The study concludes that the consumption of street‐vended barbecue chicken possesses to be a serious public health risk for consumers.

## Graphical abstract

• Control sample exhibited a better nutritional profile, and quality characteristics and also appeared to be microbiologically safe than street‐vended barbecue chicken.• Higher concentrations of highly toxic and essential metals found in barbecue chicken than their safe limits.• Results suggested the dire need of continual inspection and monitoring of food points by food regulatory authorities to implement hygienic practices from farm to fork.

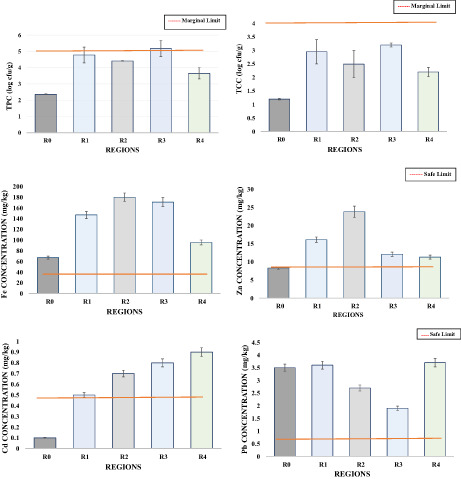

## 1 INTRODUCTION

Chicken (*Gallus gallus domesticus*) belongs to the avian species raised by humans for their eggs and meat and is considered a significant source of protein (Idowu et al., 2021; Untea et al., 2019). It is an appreciably tempting food for many societies around the globe to be used in their regional cuisines due to its good nutritional profile and ability to adjust to the local taste (Savvaidis et al., 2022). It is frequently utilized in traditional dishes where its bland flavor and texture are brought to perfection by the methods like barbecuing, frying, boiling, roasting, and steaming (Cömert & Gökmen, 2020). However, barbecuing/grilling seems to be a prominent cooking method when it comes to oxidative stability and fatty acid composition (Weber et al., 2008). Grilling appears to be speedy cooking over a direct flame while barbecuing is slow cooking for a long period of time over a direct or indirect flame (Sobral et al., 2018). The smoke produced during barbecuing comprises many phenolic compounds and formaldehyde, acts as a preservative, and provides surface dehydration which hinders bacterial growth. However, several chemical and physical changes may occur in the meat as a result of barbecuing such as the Maillard reaction which produces many flavoring and browning compounds like melanoidins that are responsible for the eccentric flavor and brown color of barbecued meat. Moreover, the high temperature of barbecue meat may cause protein denaturation resulting in a softer and palatable texture (Racovita et al., 2020).

Beyond these useful aspects of the barbequing process, many toxicants are also produced from charcoal combustion, having a deleterious impact on human health. These toxicants include carbon monoxide, greenhouse gasses, volatile organic compounds, polycyclic aromatic hydrocarbons, heterocyclic amines, hazardous trace metals, and particulate matter. However, particulate matter is found in excess because the amount of charcoal used for barbecuing increases its concentration (Niu et al., 2021; Onopiuk et al., 2022; Soeroso et al., 2021). Likewise, trace metals like Zn, Pb, Mg, Ba, Cu, Cr, Co, Cd, Ni, Mn, Se, and As are also formed throughout the burning of coal and consumed with barbecued meat (Susaya et al., 2010). The augmented feasting of chargrilled meat may mark an increased risk of intestine, pancreas, prostate, and breast cancer (Duedahl‐Olesen et al., 2015). Heavy metal toxicity is mostly determined by the concentration and type of metal. Metals such as iron (Fe), manganese (Mn), copper (Cu), zinc (Zn), and selenium (Se) are required for metabolism and regular human body functioning when consumed below permitted limits. However, some of the non‐biodegradable metals like lead, cadmium, and mercury are highly toxic to the human body even consumed in trace amounts. These non‐essential heavy metals are highly toxic and can be mutagenic, cytotoxic, and carcinogenic (El‐Wehedy et al., 2018; Rajkowska‐Myśliwiec et al., 2021).

Meat quality can also be degraded by lipid oxidation which would result in detrimental changes in meat color, flavor, and odor along with the reduction in the shelf life and nutritive value and may lead to the formation of toxic compounds. In addition, microbial growth also results in undesirable changes or spoilage of chicken meat and meat products especially due to the development of lactic acid bacteria which is greatly linked with meat spoilage (Cortinas et al., 2005; Zhang et al., 2016). Microbial contamination of chicken meat can occur at any stage from slaughtering to the processing and packaging of end product corresponding to unhygienic practices and conditions (Ogu et al., 2017; Perez‐Arnedo et al., 2021).

Barbecue chicken from street vendors poses a serious threat and safety risk to human health as it possesses a high level of aerobic microflora, *Staphylococcus aureus*, *Escherichia coli*, and *coliform* (Dagalea et al., 2021; Savvaidis et al., 2022). In developing countries like Pakistan, most of the poultry products like barbecue chicken are prepared and sold by street vendors lacking the necessary education and training about good hygienic practices, resulting in poor quality and safety status of the end product (Cardinale et al., 2005; Savvaidis et al., 2022). Furthermore, insufficient roasting/heating duration and uneven temperature distribution while cooking meat/barbecue chicken are crucial factors for infection and contamination. Street vendors usually try to reduce the shrinkage of the meat during roasting to have more profit, but at the same time, they also need to fulfill the demand and appetite of the buyers. So, they reduce the cooking time which may lead to improper cooking and unhealthy conditions (Ologhobo et al., 2010). Considering the potential of chicken meat to harbor toxic heavy metals and microbial hazards of public health significance, this study has been designed to check the nutritional quality and safety characteristics of street‐vended barbecue chicken by evaluating physicochemical, heavy metals, and microbiological safety concerns associated with barbecue chicken.

## 2 MATERIALS AND METHODS

### 2.1 Sampling plan

The control sample (R_0_) was prepared in triplicate under controlled hygienic conditions and street‐vended barbecue chicken samples were collected in triplicate from four regions of Faisalabad city including (GM Abad (R_1_), Jhang Road (R_2_), Sargodha Road (R_3_), and Satiana Road (R_4_)). The data evaluation and selection of the regions were done in consultation with the committee by keeping in view the intention behind the sampling plan to cover the main densely populated areas all around Faisalabad city. A total of 12 samples were selected to obtain randomized and representative samples from a major city in Pakistan (Faisalabad).

### 2.2 Sample preparation of barbecue chicken

All the samples were packed in sterilized polyethylene zip lock bags and brought to the laboratory with the utmost care from various street vendors according to the sampling plan under controlled conditions. All the samples were labeled on the spot with utmost care and kept in the icebox for safe transportation to the laboratory. The controlled sample was prepared after proper washing and cleaning of chicken and marinated with spices and other ingredients and kept under refrigeration conditions for 4 h. Before cooking it was brought to room temperature and each cube of chicken was threaded through a skewer. The skewers were positioned over the charcoal fire and oil was applied. The skewers were rotated periodically until the golden‐brown color was achieved. It took almost 15–20 min for the barbecue to get cooked completely.

### 2.3 Physicochemical analyses

The barbecue chicken samples were evaluated for physicochemical analyses, i.e., moisture content (hot air oven at 105°C for 24 h), ash content (muffle furnace at 600°C), fat content (Soxhlet apparatus), and protein content (Kjeldahl apparatus and factor of 6.25) by the following method explained by AOAC (2018). The pH of the barbecue chicken sample was computed by an electronic digital pH meter according to the AOAC method (2018). To determine the pH, the electrode was immersed in a slurry of barbecue samples that were prepared by homogenizing the chicken at high speed in 10 ml of distilled water.

### 2.4 Free fatty acid value

The free fatty acid (FFA) value was calculated according to the protocols used by Rahman et al. (2015). For this purpose, 5 g of sample was weighed and each sample was mixed with 30 ml chloroform and homogenized for 1 min at 10,000 rpm. Chicken particles were filtered out from the solution by using Whatman filter paper number 1 and a few drops of phenolphthalein indicator were added afterward. The filtrate was titrated with 0.01 N potassium hydroxide (KOH) solution until the red color appears.

$$\text{Free fatty acid value}\left(\%\right)=\frac{\text{Volume of titration}\times \text{Normality of}\mathrm{KOH}}{\text{Weight of sample}}\times 100.$$

### 2.5 Peroxide value

The peroxide value was calculated according to the procedures followed by Rahman et al. (2015). The 3 g of barbecue chicken sample along with 30 ml acetic acid–chloroform solution (3:2 v/v) was heated at 60°C for 3 min in order to dissolve its fat. The filter paper was used to separate the chicken particles from the permeate, and 0.5 ml of a saturated potassium iodide solution was added as an indicator. Then, the filtrate was titrated against 0.01 N sodium thiosulfate solution.

$$\text{Peroxide value}\left(\mathrm{meq}/g\right)=\frac{\text{Volume of titration}\times \text{Normality of sodium thiosulfate}}{\text{Weight of sample}}\times 100$$

### 2.6 Microbiological analyses

For the preparation of the sample, about 10 g BBQ chicken was blended with 90 ml of peptone water in a stomacher at 200 rpm for 2 min. Media for total plate count were prepared by mixing nutrient agar in distilled water. For total coliform count, media were prepared by mixing MacConkey agar in distilled water. After the preparation of both media, they were autoclaved at 121°C for 15 min at 15 psi pressure. Then, the media were poured into sterilized Petri plates until solidified and dried at 37°C in the oven and stored in the refrigerator at 2°C for further use. All the process was done in an aseptic condition.

### 2.7 Total plate count

The total plate count of barbecue chicken samples was determined by the method explained by Ogbonna et al. (2012). 0.1 ml of prepared diluted sample from each of five serial dilutions were spread on a prepared nutrient agar Petri plate using a glass spreader. These Petri plates were incubated for 48 h at 37°C.

### 2.8 Total coliform count

The total coliform count of barbecue chicken samples was calculated through the plate count method using MacConkey agar by the preparation of five serial dilutions as explained earlier and these Petri plates were incubated for 48 h at 37°C temperature (Hassan et al., 2014). Colonies that appeared on Petri plates for TPC and TCC were counted by using a digital colony counter and the final result was expressed in log cfu/g.

### 2.9 Heavy metal analyses

The level of four heavy metals was checked with Hitachi Polarized Zeeman atomic absorption spectrophotometer (AAS), Model Z‐8200, by following the conditions given in Table 1. All the samples were passed from wet digestion, and for this, 10 ml of di‐acidic mixture was prepared in a ratio of 4:1 using 70% HNO_3_ and 65% HClO_4_. The mixture was then poured into the beaker containing 2 g of weighed sample and digested over a hot plate at 80°C until it gets transparent. After digestion, the solution was filtered by using Whatman filter paper no. 42 and the remaining solution was diluted with deionized water to make up to 50 ml volume (Alturiqi & Albedair, 2012). The digested samples were analyzed through AAS against the respective standard solutions of heavy metals.

**TABLE 1:** Operational conditions used for the determination of different elements by using atomic absorption spectrophotometer

Elements	Wavelength (nm)	Slit width (nm)	Lamp current (mA)	Burner height (mm)	Oxidant gas pressure (flow rate) (kpa)	Fuel gas pressure (flow rate) (kpa)
Cadmium	228.8	1.3	7.5	5.0	160	6
Iron	248.3	0.2	10	7.5	160	6
Lead	283.3	1.3	7.5	7.5	160	7
Zinc	213.9	1.3	10.0	7.5	160	6

### 2.10 Statistical analysis

The collected data were subjected to statistical analysis using Statistics 8.1 software. One‐way analysis of variance (ANOVA) under a completely randomized design (CRD) was used according to the methods described by Montgomery (2008). In order to analyze the collected data, a completely randomized design was used and the significance of the results was evaluated.

## 3 RESULTS AND DISCUSSIONS

### 3.1 Physicochemical analyses

The mean values of moisture content pronounced that the lowest moisture (54%) was detected in control sample R_0_ (Table 2). The maximum moisture content of 62% was found in the region R_3_ followed by R_1_, R_2_, and R_4_ which exhibited moisture content of 60%, 59%, and 56%, respectively. As compared to all the samples, the control sample R_0_ showed the lowest moisture due to its preparation by upholding proper temperature. This depicted, R_0_ was more acceptable according to its safety point of view as water act as a medium for the growth of microbes and this would limit their optimum growing condition. Hasan et al. (2019) proposed the moisture content in raw boiler meat to be 75.26% ± 0.93% which was reduced upon cooking of raw meat. The moisture content was also evaluated by Oz and Yuzer (2016) in raw and barbequed meat. They reported that moisture content in raw meat was 76.91% which was reduced during barbecuing over a wire grill up to 56.98%, which is in accordance with the proposed results of the presented research.

**TABLE 2:** Physiochemical analyses of barbecue chicken samples

Regions	Moisture content (%)	Protein content (%)	Fat content (%)	Ash content (%)	pH	FFA value (%)	Peroxide value (meq/kg)
R_0_	54.00 ± 2.21^c^	32.87 ± 1.35^a^	7.25 ± 0.29^c^	1.61 ± 0.08^a^	5.60 ± 0.23^c^	1.00 ± 0.04^a^	0.63 ± 0.036^c^
R_1_	60.00 ± 2.58^ab^	28.87 ± 1.33^cd^	9.00 ± 0.41^a^	1.68 ± 0.05^a^	6.20 ± 0.26^ab^	1.32 ± 0.05^ab^	0.77 ± 0.034^b^
R_2_	59.00 ± 2.59^ab^	29.93 ± 1.32^bc^	8.23 ± 0.36^b^	1.71 ± 0.08^a^	5.95 ± 0.26^abc^	1.14 ± 0.05^c^	0.67 ± 0.029^c^
R_3_	62.00 ± 2.85^a^	26.97 ± 1.29^d^	8.29 ± 0.39^b^	1.72 ± 0.03^a^	6.30 ± 0.29^a^	1.23 ± 0.05^bc^	0.84 ± 0.039^a^
R_4_	56.00 ± 2.57^bc^	32.00 ± 1.53^ab^	8.00 ± 0.38^b^	1.66 ± 0.04^a^	5.80 ± 0.26^bc^	1.39 ± 0.06^a^	0.79 ± 0.037^ab^

*Note*: Values = Means ± SE of triplicate samples. R_0_ (Control Sample); R_1_ (Ghulam Mohammad Abad); R_2_ (Jhang Road); R_3_ (Sargodha Road); and R_4_ (Satiana Road).

The protein content of barbecue chicken from different regions R_0_, R_1_, R_2_, R_3_, and R_4_ was 32.86%, 28.86%, 29.93%, 26.96%, and 32.00%, respectively. The highest value of protein content was exhibited by the control sample it was reflected as high in nutrition. All the values of protein content favored the fact that protein content showed an appreciable increase with the loss of moisture during barbecuing. Data published in the Portuguese nutritional table point out that the protein content in raw chicken breast varies from 22% to 34.5% (Pereira & Vicente, 2013). Many researchers had also supported the fact that the increase in protein content occurred when prepared by various cooking methods like smoking, grilling, and microwaving due to the loss of moisture, which further resulted in the concentration of various amino acids (Adomeh, 2018; Akintola, 2015; Sobral et al., 2018). Liao et al. (2010) also researched the effect of various cooking methods on the proximate composition of chicken and reported the protein content in raw chicken meat to be 23.69%. While charcoal grilling of chicken breast meat for 20 min resulted in increased protein content of 32.77%, which is comparable to the present findings. Previously, protein content in the chicken meat smoked by drum Kiln method showed a very high protein content of 48.25% to 49.89% due to higher moisture losses (Adomeh, 2018).

The fat content of barbecue chicken samples revealed that a maximum amount of 9.00% of fat was found in the region (R_1_) while a minimum amount of 7.24% in R_0_. The highly significant difference between all samples could be due to non‐uniform processing conditions adopted by vendors. Fat content also varied substantially due to the application of varying amounts of oil over chicken samples during barbecuing. The present study also reinforced the fact that fat was lost during barbecuing due to its drip loss while cooking over coal. However, brushing chicken with vegetable oil during barbecuing retained the amount of fat in samples which is why a substantial amount of fat content was found in the results. The research concluded that the nutritional composition and lipid content of raw meat got highly affected by various cooking methods adopted as substantial losses in fat content of meat occurred when subjected to cooking by grilling and broiling without the addition of fat (Sobral et al., 2018). Adomeh (2018) studied the physiochemical properties of drum kiln smoked chicken thighs and estimated the fat content ranges from 6.75% to 7.11% which was near to the present outcomes. The augmented fat content was also reported by Adeyeye (2017) in the grilled meat samples due to the application of vegetable oil during processing which is comparable to the current results.

Ash is the amount of inorganic mineral that remains after the burning of water and organic components like protein and fat from food. It is the significant parameter that indicates the presence of essential minerals such as Na, K, and Ca in the food sample (Inobeme et al., 2019). The ash percentage of barbecue chicken samples showed a non‐significant difference from all regions of Faisalabad city. The ash content of street‐vended barbecue chicken samples R_1_, R_2_, R_3_, and R_4_ was 1.68%, 1.71%, 1.72%, and 1.66%, respectively, which was very close to the ash content of self‐prepared control sample R_0_ (1.61%). The findings of the present study are also close to the results obtained by also Adomeh (2018), according to which the ash content of smoked chicken thighs ranged from 1.72% to 2.00%. Similarly, Inobeme et al. (2019) reported ash contents of 1.62% in barbecue chicken, which are similar to the results of the present study.

The pH directly influences quality parameters of meat like color, tenderness, dip loss, water holding capacity, as well as microbial and oxidation rate. Many researchers discussed the fact that the low pH value of chicken meat has been linked with decreases in moisture content and tenderness along with an increase in the shelf life of meat (Mir et al., 2017; Rahman et al., 2015). The lowest value of 5.6 was observed in the control sample R_0_ trailed by R_4_, R_2_, R_1_, and R_3_ having pH of 5.8, 5.9, 6.2, and 6.3, respectively. The results depict that R_0_ has a more acidic pH as compared to all other samples, which is why it showed the lowest microbial count. All pH values of barbecue chicken samples from four regions of Faisalabad city vary significantly due to variation in the recipe and cooking parameters. The findings of Oz and Yuzer (2016) showed the pH of raw chicken to be 5.41, which upon barbecuing over wire and stone becomes 5.71 and 5.66, respectively. Previously, the pH value of charcoal grilled chicken was assessed by Liao et al. (2010) who revealed a pH value of 6.00, which was close to the current findings. The pH value of suya or grilled or barbecued meat samples sold in different areas of Nigeria was assessed which showed that the increase in the pH value contributes significantly toward the presence of different kinds of pathogenic microbes like *E*. *coli*, *S*. *aureus*, *Salmonella*, and *Shigella* as pH of order 6.8–8.0 is ideal for the microbial growth (Ogbonna et al., 2012). All these researches supported the finding of current research by declaring that changes in the pH of barbecue chicken samples significantly affect the microbial count of barbecue chicken samples.

Free fatty acids (FFA) were formed due to microbial degradation and the enzymatic activity of lipids. The oxidation of unsaturated fatty acids resulted in the development of free acidity which acts as an indicator of the deteriorative quality of oil‐containing food (Adeyeye, 2017). FFA level in the food purchased from street vendors exceeded due to prolonged food storage time and periodic heating employed to reduce the chances of spoilage and noticeable off‐flavor development that might be unacceptable to the consumer. In order to prevent the unacceptable off‐flavor, the FFA value should not exceed 1.2% (Akharaiyi, 2015; Rahman et al., 2015). The maximum FFA value (1.39%) was observed in region R_4_ and minimum was noticed in region R_2_ (1.14%), which was still higher than the FFA value for the self‐prepared sample R_0_ (1.00%).

The statistical data revealed that the FFA value of barbecue chicken samples exhibited highly significant alteration owing to a discrepancy in the storage conditions, storage time, fluctuation of freezing temperature, and improper thawing of raw chicken meat. However, the control sample showed a lower FFA value as compared to the purchased samples as it did not seem appropriate due to the extended storage conditions. Das et al. (2011) reported the acceptable limit of FFA content (1.8%) in cooked meat samples. The results of rancidity were in accordance with the current findings which revealed the FFA value of meat cooked by traditional suya smokers, electric grilling machines, and hot air ovens were 1.38%, 1.13%, and 1.01%, respectively (Adeyeye, 2017). Thus, we concluded that the meat cooked through the traditional meat smoker had higher FFA but still below the recommended values (Adeyeye, 2017). All these research‐based data supported the current research by stating that the FFA value of barbecued or smoked meat was within the suggested limit.

Lipid peroxidation is an indicator of meat quality deterioration in terms of flavor, color, texture, and nutritive value. It results in rancidity and off‐flavor due to the oxidation of lipids leading to the formation of free radicals, whose interaction with the food molecules produces ketones and aldehydes and triggers off‐flavor (Ariff et al., 2011; Domínguez et al., 2014; Rahman et al., 2015). The highest peroxide value of 0.84 meq/kg was observed in region R_3_ followed by R_4_, R_1_, and R_2_ which showed values of 0.79, 0.77, and 0.67 meq/kg, respectively (Table 2). While the lowest amount of peroxide value of 0.63 meq/kg was observed in R_0_, as it had not undergone any storage or improper thawing that was considered as the leading cause of lipid oxidation, further resulting in the development of peroxide as discussed by Ariff et al. (2011). It was concluded in research that meat cooked by grilling through a traditional meat smoker showed a higher peroxide value of 0.78 meq/kg but still below the recommended values of between 20 and 40 mgeq/kg for rancid taste (Adeyeye, 2017).

### 3.2 Microbiological analyses

**FIGURE 1:**
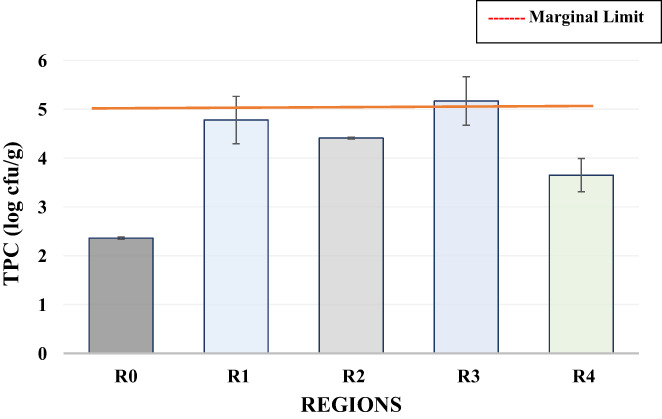
Variation in the TPC (log cfu/g) of barbecue chicken samples

The safety status of barbecue chicken was evaluated by total plate count (TPC) and total coliform count (TCC). Chicken meat is the ideal medium of growth for food‐borne pathogens as well as spoilage‐causing microbes at any stage of food preparation (Mead, 2004). Total plate count between 4 and 5 log cfu/g was considered marginal, while TPC of more than 5 log cfu/g was considered unsatisfactory (Manguiat & Fang, 2013). Statistical analysis of the TPC of barbecue chicken samples showed that the TPC of barbecue chicken from all regions of Faisalabad city varied significantly due to varied hygiene conditions followed by the processor. The lowest TPC value of 2.36 log cfu/g was found in self‐prepared sample R_0_ followed by 3.65 log cfu/g in R_4_, 4.41 log cfu/g in R_2_, 4.78 log cfu/g in R_1_, and 5.71 log cfu/g in R_3_ (Figure 1). The TPC of R_3_ was considered unsatisfactory indicating poor sanitation and hygiene practices. The results of this study are comparable to the research of Kigigha et al. (2015); Manguiat and Fang (2013); and Ologhobo et al. (2010).

**FIGURE 2:**
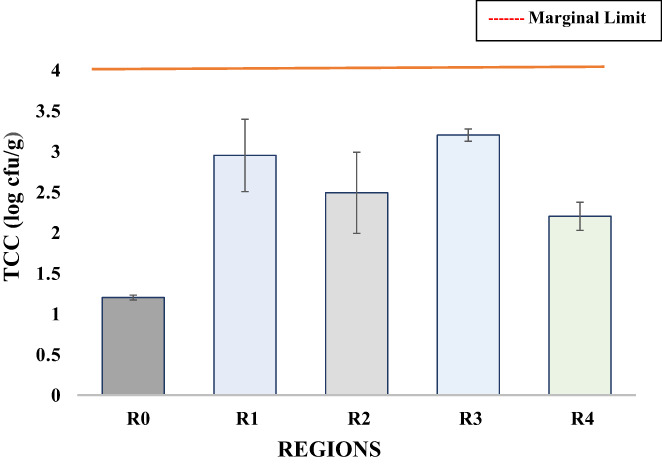
Variation in the TCC (log cfu/g) of barbecue chicken samples

Coliform including fecal coliform or *E*. *coli* is facultative anaerobia, acts as an indicator of meat quality, and its occurrence exhibits the adoption of poor hygiene practices or unsanitary conditions during food processing (Jaja et al., 2018; Odwar et al., 2014). A coliform count greater than 4 log cfu/g is considered unsatisfactory, while between 2 and 4 log cfu/g is marginal (Manguiat & Fang, 2013). The statistical results indicate that the TCC of barbecue chicken samples differ significantly and vary from 1.20 to 3.20 log cfu/g (Figure 2). Minimum TCC was found in self‐prepared R_0_, which confirmed that it was prepared under good hygiene and sanitary conditions. While the highest TCC was exhibited by region R_3_. The results of the TCC count of all the street‐vended barbecue chicken samples were not satisfactory but still within the marginal limit, while the control sample showed satisfactory results for TCC. The findings of this research were in accordance with Kigigha et al. (2015); Manguiat and Fang (2013); Ologhobo et al. (2010).

### 3.3 Heavy metal analyses

The heavy metal most probably accumulated in meat resulting in a source of heavy metal, when consumed above the permitted limit, has a negative effect on human well‐being (Joyce et al., 2016). An Atomic Absorption Spectrophotometer (Model, Z‐8200) was used to evaluate the level of four heavy metals (Fe, Zn, Cd, and Pb) in the consumed barbecue chicken samples.

**FIGURE 3:**
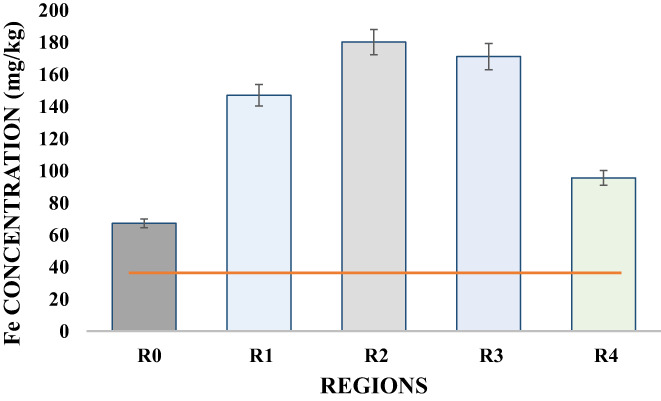
Variation in the Fe concentration (mg/kg) of barbecue chicken samples

Iron is required by the body in small concentrations for normal growth, proper functioning of the immune system, DNA synthesis, etc. However, the increased concentration of iron is likely unsafe for children and may result in serious complications due to iron toxicity (Ujowundu et al., 2014). The highly significant difference in all mean values showed iron content of 146.90, 180.00, 171.00, and 95.40 mg/kg in regions R_1_, R_2_, R_3_, and R_4_, respectively (Table 3; Figure 3). A minimum concentration of 67 mg/kg was found in the R_0_. Results obtained in this study were closely related to the study of Joyce et al. (2016) who reported that the iron content in the meat promptly increased by grilling. However, no other cooking methods resulted in an abrupt increase in the iron concentration than grilling. This could be related to the fact that the meat comes in direct contact with the iron skewers and few of its particles get into the meat during the process of grilling or smoking which would further result in an increased level of iron than acceptable level. Similar increase in the concentration of iron was observed in the current results which was found to be much greater than recommended levels of iron (Fe) concentration for human consumption (4.49 ppm to 15.0 ppm) (Joyce et al., 2016).

**TABLE 3:** Heavy metal concentration of barbecue chicken samples

Regions	Fe (mg/kg)	Zn (mg/kg)	Cd (mg/kg)	Pb (mg/kg)
	Safe limit (4.49–15.0)	Safe limit (0.41–5.0)	Safe limit (0.33)	Safe limit (0.01–0.38)
R_0_	67.10 ± 2.751^d^	8.30 ± 0.340^d^	0.10 ± 0.004^c^	3.50 ± 0.14^a^
R_1_	146.90 ± 6.757^b^	16.10 ± 0.741^b^	0.50 ± 0.022^d^	3.60 ± 0.155^a^
R_2_	180.00 ± 7.920^a^	23.80 ± 1.575^a^	0.70 ± 0.031^c^	2.70 ± 0.119^b^
R_3_	171.00 ± 8.208^a^	12.10 ± 0.581^c^	0.80 ± 0.037^b^	1.90 ± 0.087^c^
R_4_	95.40 ± 4.579^c^	11.30 ± 0.542^c^	0.90 ± 0.041^a^	3.70 ± 0.170^a^

*Note*: Values = Means ± SE of triplicate samples. R_0_ (Control Sample); R_1_ (Ghulam Mohammad Abad); R_2_ (Jhang Road); R_3_ (Sargodha Road); and R_4_ (Satiana Road).

^a–d^ Means within a column with different superscripts differ significantly.

**FIGURE 4:**
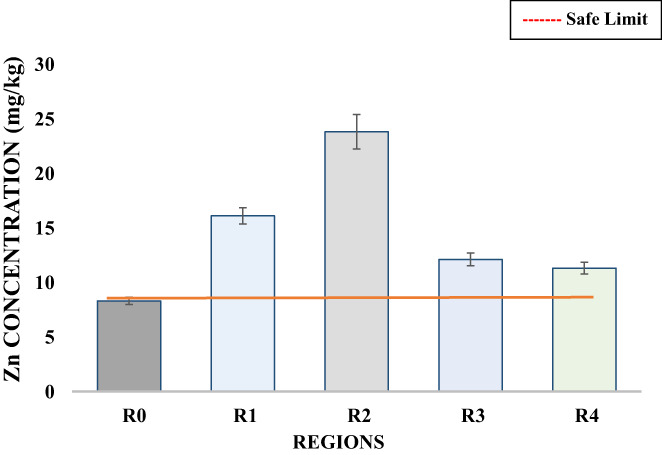
Variation in the Zn concentration (mg/kg) of barbecue chicken samples

Zinc is also among the nutritionally essential trace element that is required essential by the body in minute quantities for a healthy life. Chicken meat, beef, and fish are important sources of zinc. Consumption of an increased amount of zinc may result in reduced Ca absorption further causing anemia while consumption of a very high amount was unsafe and may cause prostate cancer (Oloruntoba & Nathaniel, 2019). The safe limit for Zn is 0.41–5 ppm (Joyce et al., 2016). Moreover, a higher concentration of zinc was also reported to result in zinc intoxication and death of experimental animals. The zinc content found in the barbecue chicken samples was 8.30 mg/kg in R_0_, 16.10 mg/kg in R_1_, 23.80 mg/kg in R_2_, 12.10 mg/kg in R_3_, and 11.30 mg/kg in R_4_ (Table 3; Figure 4). The highest concentration of zinc was found in R_2_ and the lowest value was shown by R_0_. Statistical analysis of zinc content in all barbecue chicken samples showed a highly significant difference between all samples. Results obtained were closely related to the work of Mariam et al. (2004), who reported the concentration of zinc in poultry meat sold in Pakistan to be 28.52 ppm. Joyce et al. (2016) also reported a Zn concentration of 6.97 ppm in a grilled meat sample which was also close to the Zn concentration in a few of the current samples. While much higher concentration of 20.80 mg/kg was also observed in freshly processed chicken samples was also found to be in accordance with current results (Ujowundu et al., 2014).

**FIGURE 5:**
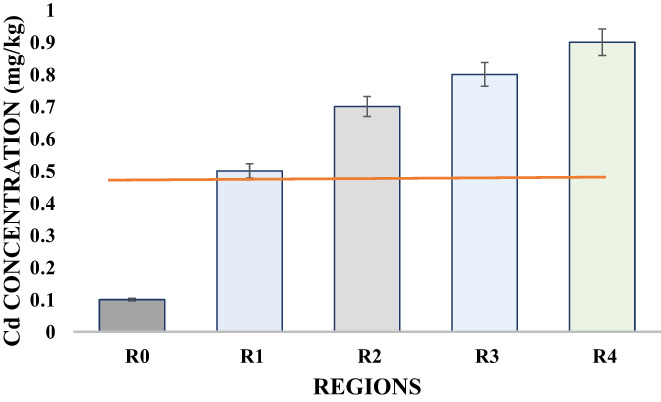
Variation in the Cd concentration (mg/kg) of barbecue chicken samples

Cadmium is one of the most dangerous heavy metals that is not required by the body to perform its biological functions and can also enter the human body through contaminated food items (Hashemi et al., 2017). Safe limit for cadmium is 0.33 mg/kg (Joyce et al., 2016). The concentration of cadmium in barbecue chicken samples from all regions of Faisalabad city varied significantly. The Cd concentration was noticed as 0.10, 0.50, 0.70, 0.80, and 0.90 mg/kg in R_0_, R_1_, R_2_, R_3_, and R_4_, respectively (Table 3; Figure 5). The minimum content of Cd was detected in control (R_0_) which was within the safe limit while all other samples brought from different regions of Faisalabad city showed much higher concentrations of cadmium. The concentration of Cd in the samples from regions GM Abad (R_1_), Jhang Road (R_2_), Sargodha Road (R_3_), and Satiana Road (R_4_) was detected to be much higher than that of the safe limit of 0.33 mg/kg as the process of barbecuing over coal found to be the source of contamination by heavy metals. It was concluded that the grilling process intoxicates the samples with heavy metals (Nnaji & Ogbuewu). Similarly, Ujowundu et al. (2014) also discussed in their research that preparing meat by smoking under wood or ashes resulted in a significant increase in the heavy metals concentration. Previously, Mariam et al. (2004) reported the higher concentration of cadmium in chicken meat sold in Pakistan and reported it to be higher than the permissible limit stated by FAO/WHO due to environmental concerns. All these research data strongly supported the results of the current research.

**FIGURE 6:**
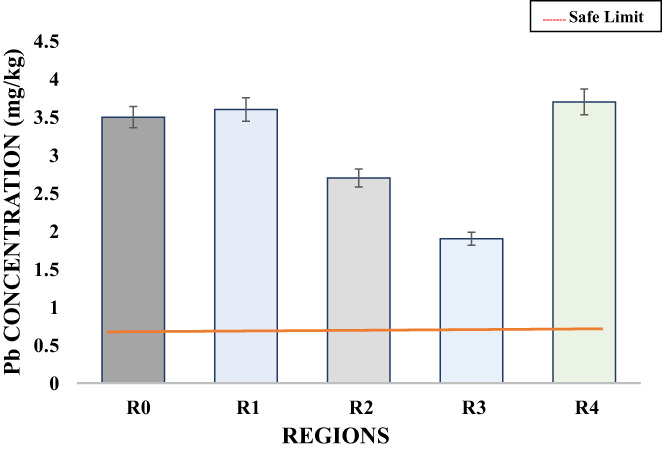
Variation in the Pb concentration (mg/kg) of barbecue chicken samples

Lead is an exceedingly noxious heavy metal and pediatric poison that wield antagonistic effects on the brain, bones, kidney, and thyroid gland. It was numbered second among the list of 20 hazardous substances according to the data presented by Agency for Toxic Substances and Disease Registry (ATSDR) (Ujowundu et al., 2014). The safe limit of lead for human consumption without causing adverse effects ranges from 0.01 to 0.38 ppm (Joyce et al., 2016). The results showed that the concentration of lead in R_3_ was 1.90 mg/kg followed by the concentration of 2.70 mg/kg, 3.50 mg/kg, 3.60 mg/kg, and 3.70 mg/kg in the R_2_, R_0_, R_1_, and R_4_, respectively (Table 3, Figure 6). Lead concentration in barbecue chicken samples from all regions showed a highly significant difference and they were much higher than the recommended safe limit in all samples. The higher concentration of lead in all samples was induced during barbecuing over coal or may be due to environmental pollution caused by the vehicles moving alongside vending shops or maybe induced primarily due to contaminated feed and water. All these reasons for heavy metal contamination were discussed by Nnaji and Ogbuewu (2017) and Ujowundu et al. (2014). The results of the current study were also in accordance with the study of Mariam et al. (2004), who reported a higher level of lead 3.1 ppm in poultry meat sold in Pakistan than reported by Abdel‐Hassan et al. (2000); and Nnaji & Ogbuewu; Ujowundu et al. (2014) due to predominant environmental pollution. (Ujowundu et al., 2014) also reported the an increased concentration of 3.70 in long long‐processed chicken. All these research evidences support the results of the current research study.

## 4 CONCLUSIONS

The current study revealed the quality, microbial safety, and heavy metal profile of street‐vended barbecue chicken sold in Faisalabad, Pakistan. The control sample exhibited a much better nutritional profile, quality characteristics, and also appeared to be microbiologically safe as it showed satisfactory TPC and TCC (prepared by adopting good hygienic and sanitary conditions) while the highest TPC and TCC were observed in the samples from Sargodha Road (R_3_). The concentration of essential metals (Fe and Zn) and highly toxic heavy metals (Pb and Cd) ranged from 67.10–180 (Fe), 8.30–35.80 (Zn), 1.90–3.70 (Pb), and 0.10–0.90 (Cd), respectively. All the metals showed alleviated levels in overall samples except for the Cd which was within the safe limit only in the control sample. The street‐vended barbecue chicken samples exhibited eminent heavy metals toxicity (Pb and Cd). The results of the current study manifested that the microbiological and heavy metal profile of street‐vended barbecue chicken is questionable and thus requires strict surveillance regarding food safety standards. Results suggested the dire need for continual inspection and monitoring of food points by food regulatory authorities to implement hygienic practices from farm to fork. Effective measures include training of food personnel, good manufacturing practices (GMP), and internal audits.

## CONSENT TO PARTICIPATE

All authors extend consent to participate as co‐authors.

## CONFLICT OF INTEREST

The authors have no conflict of interest.

## Data Availability

The dataset supporting the conclusions of this article is included within the article
